# Diagnostic accuracy of MRI and US for identifying acute rejection after allogeneic kidney transplantation: a systematic review and meta-analysis

**DOI:** 10.1016/j.clinsp.2026.100999

**Published:** 2026-05-18

**Authors:** Rong Qian, Ya-fei Zhang, Xin-yan Zhou, Yi-hua Bai, Dong Chen, Tao Wu, Guo-dong Zhang, Wan Shen, Sha-sha Bao, Na Tan, Yi Lu, Cheng-de Liao, Zhi-qiang Ouyang

**Affiliations:** a Department of Radiology, Yan’ an Hospital Affiliated to Kunming Medical University, (Kunming Yan’ an Hospital), Kunming, China; b Key Laboratory of Tumor Immunological Prevention and Treatment of Yunnan Province, Kunming, China; c Department of Urology, The First People's Hospital of Kunming, Kunming, China; d Department of Nephrology, The Second Affiliated Hospital of Kunming Medical University, Kunming, China; e Department of Ultrasound, Yunnan Cancer Hospital (The Third Affiliated Hospital of Kunming Medical University), Kunming, China; f Department of Colorectal Surgery, Yunnan Cancer Hospital (The Third Affiliated Hospital of Kunming Medical University), Kunming, China; g Bidding and Procurement Office, Yunnan Cancer Hospital (The Third Affiliated Hospital of Kunming Medical University), Kunming, China; h Department of Radiology, The First Affiliated Hospital of Kunming Medical University), Kunming, China

**Keywords:** Kidney transplantation, Magnetic resonance imaging, Ultrasound imaging, Diagnosis accuracy, Meta-analysis

## Abstract

**Objectives:**

To evaluate the diagnostic performance of multiparametric Magnetic Resonance Imaging (MRI) and Ultrasound (US) for detecting Acute Immune Rejection (AIR) after kidney transplantation through a systematic review and meta-analysis.

**Methods:**

Five electronic databases were systematically searched up to May 30, 2025. Eligible studies included kidney transplant recipients assessed with noninvasive imaging for AIR and reporting diagnostic accuracy. Two reviewers independently performed study selection, data extraction, and quality assessment using the Quality Assessment of Diagnostic Accuracy Studies-2 (QUADAS-2). Given the heterogeneity of imaging modalities, MRI and US studies were analyzed separately, and subgroup analyses and meta-regression were performed to explore sources of heterogeneity.

**Results:**

Eighteen studies (1140 cases; 494 AIR and 646 non-AIR) were included. For MRI, the pooled sensitivity, specificity, and Area Under the Curve (AUC) were 0.89, 0.91, and 0.96, respectively. For US, the pooled sensitivity, specificity, and AUC were 0.79, 0.89, and 0.90, respectively. Substantial between-study heterogeneity was observed. Meta-regression and subgroup analyses suggested that heterogeneity was associated with reference standard and imaging timing.

**Conclusion:**

MRI and US demonstrate promising accuracy for detecting AIR after kidney transplantation. However, the available evidence remains limited in scale, and substantial heterogeneity exists across studies. Therefore, MRI and US should be considered adjunctive tools rather than replacements for histopathological biopsy. Further large-scale, prospective studies are required to validate their clinical utility.

## Highlights

• MRI and ultrasound detect acute rejection after a kidney transplant.
• Meta-analysis shows a pooled AUC of 0.92.
• Diagnostic performance varies with timing and reference standard.
• Noninvasive imaging may reduce reliance on biopsy.
• Multimodal imaging supports early detection and patient management.

## Introduction

Allogeneic kidney transplantation remains the most effective therapeutic option for patients with end-stage renal disease. However, Acute Immune Rejection (AIR) continues to be a major cause of graft dysfunction and even allograft loss.^1^^,^^2^ AIR often occurs within days to months after transplantation, with an insidious onset and rapid progression. Without timely recognition and intervention, it can lead to irreversible renal parenchymal injury and eventual graft failure. At present, percutaneous renal biopsy, interpreted according to the Banff classification, is still regarded as the diagnostic gold standard for AIR.^3^^,^^4^ Nevertheless, as an invasive procedure, biopsy carries inherent risks ‒ including hemorrhage, local infection, and procedure-related graft injury ‒ and is unsuitable for frequent, dynamic monitoring, limiting its routine clinical application. Consequently, there is an urgent need for a non-invasive, repeatable, and quantitative imaging approach to facilitate the early detection of AIR, which has become a research priority in post-transplant functional surveillance.

**Fig. 1:**
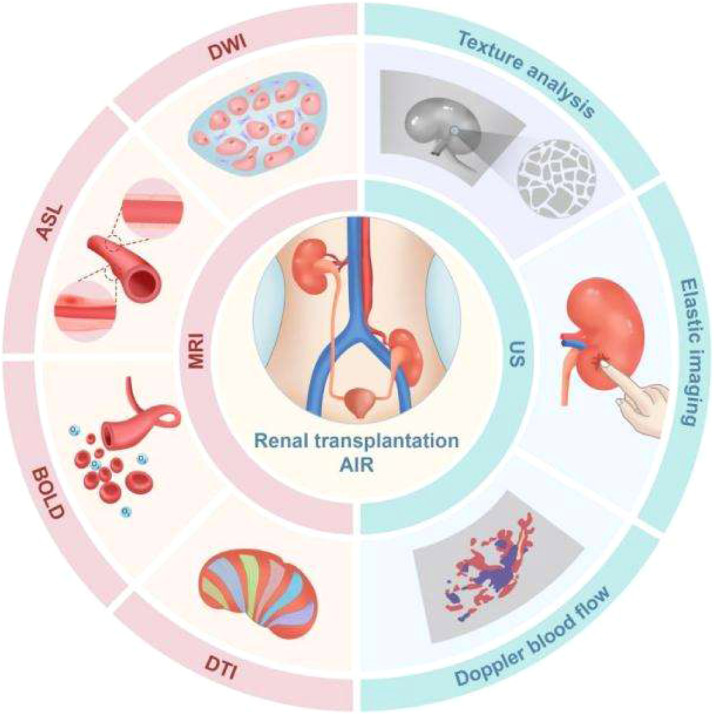
Overview diagram of diagnosing acute immune rejection after kidney transplantation by MRI and US. The blue semicircle on the left represents Ultrasound (US) imaging techniques, including texture analysis, ultrasound elastography, and color Doppler flow imaging. The semicircle on the right represents Magnetic Resonance Imaging (MRI) techniques, including Diffusion Weighted Imaging (DWI), Arterial Spin Labeling (ASL) imaging, Blood Oxygen Level Dependent (BOLD) imaging, and Diffusion Tensor Imaging (DTI). The central circle shows a schematic diagram of Acute Immune Rejection (AIR) after kidney transplantation.

In recent years, Magnetic Resonance Imaging (MRI) and Ultrasonography (US) have emerged as promising non-invasive modalities for evaluating renal allograft function (Fig. 1).^5^, ^6^, ^7^ MRI, particularly when employing multiparametric sequences such as Blood Oxygen Level-Dependent imaging (BOLD), Arterial Spin Labeling (ASL), Diffusion Tensor Imaging (DTI), and Intravoxel Incoherent Motion Imaging (IVIM), can provide comprehensive assessments of graft perfusion, oxygenation, microstructural integrity, and filtration function without the need for contrast agents;^8^, ^9^, ^10^ Likewise, advanced US techniques, including Shear Wave Elastography (SWE), Texture Analysis (TA), and color Doppler-derived hemodynamic indices such as Resistive Index (RI), have been widely applied to monitor changes in tissue stiffness and vascular resistance.^11^^,^^12^ Notably, such imaging biomarkers often exhibit abnormalities during the early stages of AIR, potentially enabling timely diagnosis before overt functional decline. Although multiple studies have reported the potential diagnostic value of MRI and US for detecting AIR, considerable heterogeneity exists among published findings due to differences in imaging protocols, parameter settings, and diagnostic criteria.^13^ Currently, no consensus has been reached on the optimal imaging modality or parameter combination with the highest sensitivity and specificity for AIR detection. Moreover, many studies are limited by small sample sizes, resulting in reduced statistical power and restricting the generalizability of their conclusions.

To address these gaps, the authors conducted a systematic review and meta-analysis to comprehensively evaluate the diagnostic accuracy of MRI and US in detecting AIR, and to compare the performance of different imaging modalities and quantitative parameters. The present study represents, to the best of our knowledge, the first comprehensive meta-analysis synthesizing evidence on the accuracy of MRI and US for noninvasive detection of post-transplant acute rejection. To ensure methodological rigor, the authors applied strict inclusion criteria, requiring pathological confirmation of AIR or non-AIR based on the Banff classification, and followed the PRISMA-DTA reporting guidelines to transparently present this study’s analytic process and findings.

## Methods

The study protocol was prospectively registered in the International Prospective Register of Systematic Reviews (PROSPERO; registration number CRD420251052942, available at: https://www.crd.york.ac.uk).

### Literature search strategy

A comprehensive literature search was conducted in five major international databases (PubMed, Embase, Web of Science, Cochrane Library, and ClinicalTrials.gov) from their inception to May 30, 2025 to identify potentially relevant studies. Search strategies combined relevant keywords and Medical Subject Headings (MeSH) terms, including: ‘kidney transplantation’, ‘graft rejection’, ‘magnetic resonance imaging’, and ‘ultrasonography’. The detailed search formulas are provided in the Appendix. No language restrictions were applied to maximize the inclusion of eligible studies. In addition, the reference lists of relevant articles were manually screened to identify any additional studies that might have been missed by the electronic search.

### Literature selection

Inclusion criteria were as follows: (I) Patients who underwent allogeneic kidney transplantation; (II) Studies utilizing MRI and/or US to assess allograft function; (III) Diagnosis confirmed by percutaneous biopsy as the reference standard.

Exclusion criteria included: (I) Non-peer-reviewed publication types such as reviews, meta-analyses, clinical guidelines, case reports, conference abstracts, letters to the editor, or commentaries; (II) In vitro or animal studies; (III) Insufficient data preventing construction or derivation of a diagnostic 2 × 2 contingency table, or inability to obtain absolute numbers of True Positives (TP), False Positives (FP), True Negatives (TN), and False Negatives (FN); (IV) Studies focusing on non-acute rejection or chronic rejection rather than acute immune rejection.

To minimize subjective bias, study selection was independently performed by two reviewers (Rong Qian and Xin-yan Zhou), with disagreements resolved by a third reviewer (Zhi-qiang Ouyang).

### Data extraction and quality assessment

The quality assessment was conducted using an 18-item checklist. Two reviewers (Qian Rong and Zhou Xinyan) independently performed the evaluation employing RevMan software (version 5.4, Cochrane; https://training.cochrane.org) based on the Quality Assessment of Diagnostic Accuracy Studies-2 (QUADAS-2) tool. QUADAS-2 assigns risk of bias judgments (‘low’, ‘high’ or ‘unclear’) for each domain according to responses (‘yes’, ‘no’ or ‘unclear’) to signaling questions within that domain. Specifically, a domain is rated as low risk of bias if all signaling questions are answered ‘yes’ and high risk of bias if all are answered ‘no’. Any discrepancies in scoring were resolved through consensus discussion.

Data extraction was performed using a predefined standardized form, which included the following components: (I) Basic study characteristics (author name, journal, publication year, country, study design, and study period); (II) Patient demographics and clinical features (sample size, rejection type, pathological findings); (III) Imaging diagnostic protocols (imaging modality, timing, sequences, quantitative parameters, and cut-off values); and (IV) Absolute counts of TP, FP, TN, and FN. The data extraction form was pilot tested on three randomly selected studies prior to formal use. Subsequently, one reviewer (Rong Qian) extracted all data items, which were then independently verified by a second reviewer (Zhiqiang Ouyang) to ensure accuracy. For each study, the imaging parameter with the highest diagnostic accuracy was selected. According to the extraction protocol, quantitative parameters were prioritized over qualitative parameters, combined parameters, and texture analyses.

### Statistical analysis

First, the threshold effect was assessed using Meta-DiSc software (version 1.4; https://meta-disc.software.informer.com). In brief, the presence of a threshold effect was evaluated by calculating the Spearman correlation coefficient between the logit of sensitivity and the logit of 1-specificity. A Spearman correlation coefficient greater than 0.8, combined with a p-value <0.05, was considered indicative of a significant threshold effect.

Subsequently, pooled sensitivity, specificity, Positive Likelihood Ratio (PLR), Negative Likelihood Ratio (NLR), Diagnostic Odds Ratio (DOR), and their corresponding 95% Confidence Intervals (95% CIs) were calculated using a random-effects model in Stata software (MP 18.0; StataCorp; https://www.stata.com). Corresponding forest plots were generated to visualize the results. Additionally, a hierarchical logistic regression model was employed to construct the Summary Receiver Operating Characteristic (SROC) curve and calculate the Area Under the Curve (AUC) to assess overall diagnostic accuracy.

Inter-study heterogeneity was assessed using the Q test and quantified with the I² statistic. According to Higgins et al.,^14^ *I*^2^ values of 25%, 50%, and 75% represent low, moderate, and high heterogeneity, respectively. Potential sources of heterogeneity in this meta-analysis included sample size (dichotomized by the median of included studies), study design, type of immune rejection, reference standard, imaging modality, timing of imaging, and published year. Meta-regression and subgroup analyses were conducted to explore and explain the origins of heterogeneity based on these variables. Additionally, sensitivity analyses were performed to evaluate the robustness of the results. Publication bias was assessed visually using funnel plots. The overall pooled results are presented only to demonstrate the global trend and are not used as the basis for cross-modality comparison.

## Results

### Study selection

**Fig. 2:**
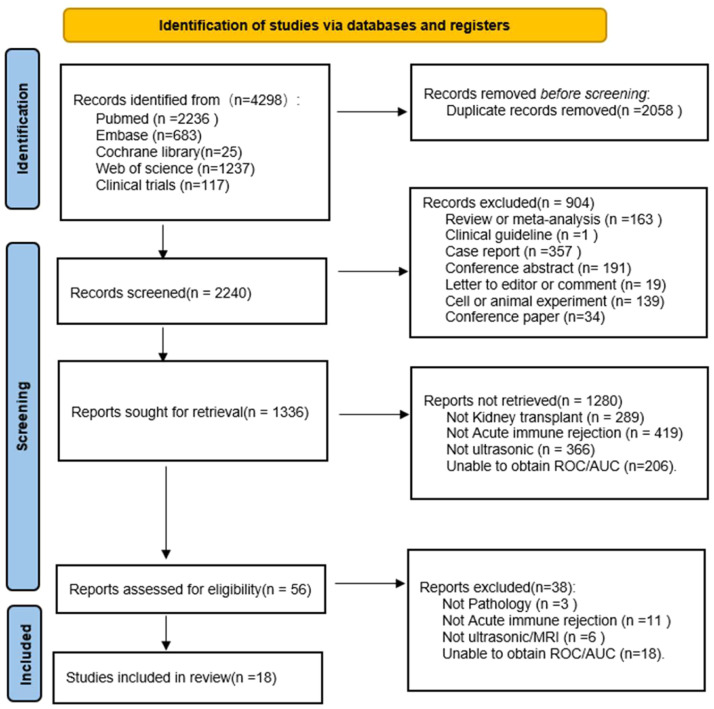
PRISMA flowchart of included studies. PRISMA, Preferred Reporting Items for Systematic Review and Meta-Analysis.

Fig. 2 illustrates the PRISMA flow diagram of this meta-analysis. A total of 4298 records were identified through database searching. Initially, 2058 duplicate records were removed, and an additional 904 records were excluded based on title and abstract screening. Of the remaining 1336 records assessed for eligibility, 1280 were excluded for not meeting the predefined inclusion criteria. Subsequently, a full-text review was conducted on 56 articles, resulting in 18 studies being included in the final systematic review and meta-analysis. Collectively, these studies encompassed 1140 patients, of whom 494 (43.3%) experienced acute immune rejection, and 646 (56.7%) did not. Due to repeated imaging examinations performed on the same patients in some studies^15^^,^^16^ the total number of included patients does not exactly correspond to the sum of AIR and non-AIR cases. Sample sizes across studies ranged from 17 to 268, with a median of 50 patients. The overall male-to-female ratio was approximately 2.4:1. All patients were definitively diagnosed by renal biopsy, comprising 494 AIR cases and 646 non-AIR cases.

### Study characteristics

Table 1 summarizes the key characteristics of the 18 studies included in this analysis. Among them, six studies employed a prospective design. All studies used the Banff criteria as the reference standard for diagnosing AIR of the renal allograft. Specifically, one study combined histopathological biopsy, serum creatinine changes, and clinical course to determine rejection status; five studies integrated histopathology with clinical treatment response; and the remaining twelve studies relied solely on histopathological biopsy as the reference standard. Ultrasound imaging was the most commonly used modality among the included studies. Additionally, three studies^17^, ^18^, ^19^ utilized Diffusion-Weighted Imaging (DWI) sequences to assess restricted water molecule diffusion in the renal parenchyma, and one study^17^ employed BOLD imaging to evaluate renal cortical and medullary oxygenation status. All studies reported varying degrees of imaging correlates associated with AIR. Additional study characteristics are detailed in Supplementary Table S1.

**Table 1:** Characteristics of studies included in the meta-analysis.

N°	First author	Journal	Period of study	Year	Nation	Study design	ICC	Reference standard	Interval of imaging scan	Imaging type
1	Z. Zhang^25^	Acad Radiol	2020/7–2023/4	2024	China	Retrospective	Yes	Pathology	Pre-biopsy	US
2	C. Yang^20^	Ultrasound in Medicine and Biology	2011/1–2014/7	2016	China	Prospective	Yes	Pathology	≤1-day post-biopsy	US
3	J.Xu^19^	Journal of Zhejiang university	NR	2010	China	Prospective	Yes	Pathology	≤5-days pre-/post-biopsy	MRI
4	M.S. van Leeuwen^46^	European Journal of Radiology	NR	1992	Netherlands	Retrospective	NR	Pathology & Therapeutic response	Postoperative weeks 1‒3: twice weekly	US
5	Werner L. Swobodnik^31^	Ultrasound in Medicine and Biology	1982/10–1984/1	1986	Germany	Prospective	NR	Pathology & Therapeutic response	≤1-day post-transplant	US
6	Manrita K. Sidhu^47^	Journal of Clinical Ultrasound	1994/5–1996/10	1999	America	Retrospective	Yes	Pathology	≤1-day pre-biopsy	US
7	Mohamed Shehata^17^	Medical Physics	2016/6–2019/6	2020	America and Egypt	Retrospective	Yes	Pathology	≤2-days post-biopsy	MRI
8	Gregory J. Raiss^48^	Journal of Ultrasound in Medicine	NR	1986	America	Retrospective	Yes	Pathology	≤1-day post-transplant	US
9	M.R.N. Nampoory^49^	Transplantation Proceedings	1994/1–1995/12	1997	Kuwait	Retrospective	Yes	Pathology & Therapeutic response	≤1-day post-biopsy	US
10	K.-P. Grigat^16^	Transplant International	NR	1989	Germany	Retrospective	Yes	Pathology	NR	US
11	Nitin P. Ghonge^21^	Radiology	2014/10–2016/3	2018	India	Prospective	NR	Pathology & Therapeutic response	NR	US
12	Paula García Barquín^23^	Diálisisy Trasplante	2010/8–2013/4	2015	Spain	Retrospective	Yes	Pathology	NR	US
13	Michael J. Germain^15^	Clinical Transplantation	NR	1992	America	Retrospective	Yes	Pathology & Therapeutic response & Serum creatinine variation	Every 2‒3 days post-transplant	US
14	Mathis P. Frick^30^	Radiology	NR	1981	America	Retrospective	Yes	Pathology	≤24-hours pre-biopsy	US
15	Abeer Mohammed Abd El-Motaal.^22^	Egyptian Journal of Radiology and Nuclear Medicine	2017/10–2019/1	2019	Egypt	Prospective	Yes	Pathology	≤1-day post-transplantation	US
16	R Datta^24^	Australasian Radiology	2001/1–2002/4	2005	India	Prospective	NR	Pathology	≤3-days pre-biopsy	US
17	Hisham Abdeltawab^18^	Scientific Reports	NR	2019	America, Egypt and UAE	Retrospective	NR	Pathology & Therapeutic response	NR	MRI
18	Ali Abbasian Ardakani^50^	Iranian Journal of Kidney Diseases	2012/7–2014/7	2014	Iran	Retrospective	NR	Pathology	NR	US

### Quality assessment

**Fig. 3:**
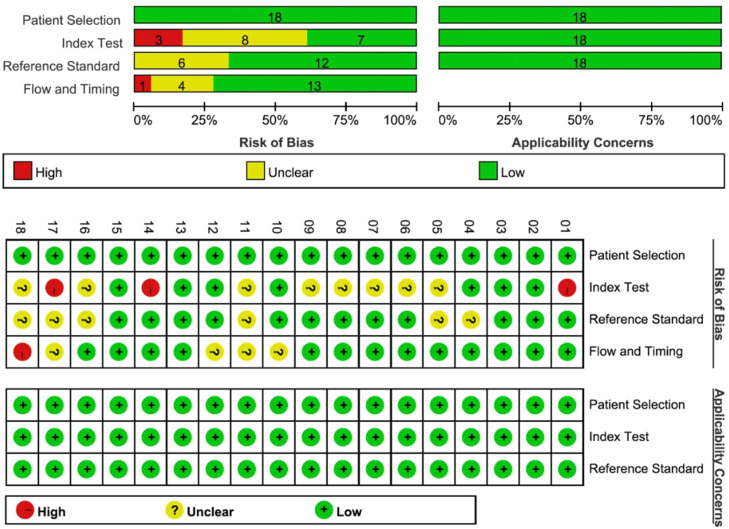
Risk of bias and applicability concern graph for each included study after arbitration between reviewers. -, high risk; ?, unclear risk; +, low risk.

The results of the QUADAS-2 quality assessment for the included studies are presented in Fig. 3. Both independent reviewers consistently noted that most studies did not clearly report whether diagnostic thresholds were pre-specified before study commencement, whether image interpretation or reference standard assessment was performed in a blinded manner, or whether an appropriate interval existed between imaging and reference standard application. Consequently, eight studies were rated unclear regarding index test bias, six studies regarding reference standard bias, and four studies had unclear risk related to flow and timing.

### Threshold effect evaluation

The correlation analysis revealed a Spearman correlation coefficient of −0.417 (*p* = 0.108) between the logit of sensitivity and the logit of 1-specificity, indicating no significant threshold effect among the included studies. This suggests that the observed heterogeneity was not attributable to threshold effects, thereby confirming the feasibility and validity of conducting the subsequent meta-analysis.

### Diagnostic accuracy

**Fig. 4:**
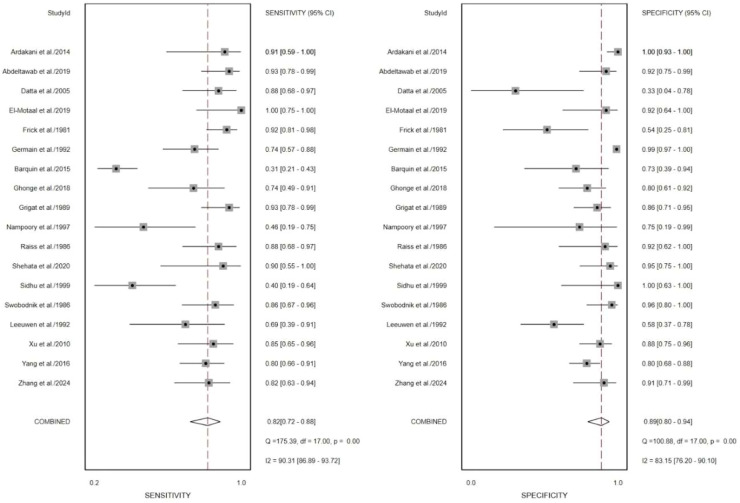
Forest plot of sensitivities and specificities of the included studies.

Prior to pooling the diagnostic performance metrics, the authors selected the most representative quantitative parameters from each study in accordance with the predefined data extraction criteria. Across all included studies, the most effective parameters were Shear Wave Stiffness (SWS) values reported in three studies,^20^, ^21^, ^22^ Resistive Index (RI) in six studies,^15^^,^^20^, ^21^, ^22^, ^23^, ^24^ Pulsatility Index (PI) in four studies,^16^^,^^24^^,^^25^ ADC values in three studies,^17^, ^18^, ^19^ and R2 values in one study^17^ (Table 1). The summary diagnostic performance of the 18 included studies is presented in Table 2. The pooled sensitivity and specificity were 0.82 (95% CI 0.72‒0.88) and 0.89 (95% CI 0.80‒0.94), respectively (Fig. 4). The combined Positive Likelihood Ratio (PLR) and Negative Likelihood Ratio (NLR) were 7.60 (95% CI 3.97‒14.54) and 0.21 (95% CI 0.13‒0.33), respectively, yielding a Diagnostic Odds Ratio (DOR) of 36.78 (95% CI 14.53‒93.14) (Figs. S1 and S2). The summarized hierarchical Summary Receiver Operating Characteristic (SROC) curve (Fig. 8) demonstrated an Area Under the Curve (AUC) of 0.92 (95% CI 0.89‒0.94), approaching 1.0, indicating that both MRI and US are highly effective imaging modalities for detecting AIR after kidney transplantation.

**Table 2:** Meta-analysis results of sample size, study design, gold standard, imaging methods, imaging series, check interval and published year.

Subgroup	Number	Pooled sensitivity (95% CI)	p^1^	Pooled specificity (95% CI)	p^2^
Sample size					
< 50 patients	7	0.79 (0.67‒0.91)		0.85 (0.71‒0.98)	
≥ 50 patients	9	0.87 (0.80‒0.94)	0.41	0.89 (0.81‒0.97)	0.62
Study design					
Retrospective	10	0.80 (0.71‒0.90)		0.89 (0.80‒0.97)	
Prospective	6	0.89 (0.81‒0.97)	0.50	0.85 (0.73‒0.98)	0.19
Gold standard					
Pathology and therapeutic response	5	0.78 (0.63‒0.92)	0.02	0.85 (0.70‒0.99)	0.22
Pathology	11	0.86 (0.79‒0.94)	.	0.89 (0.81‒0.97)	.
Imaging methods					
US	13	0.82 (0.75‒0.90)		0.86 (0.77‒0.94)	
MRI	3	0.90 (0.80‒1.00)	0.96	0.93 (0.83‒1.00)	0.76
Check interval					
> 1 day pre-/post-	8	0.81 (0.71‒0.92)	0.02	0.87 (0.76‒0.98)	0.28
≤ 1 day pre-/post-	8	0.87 (0.78‒0.95)	.	0.88 (0.79‒0.97)	.
Published year					
After 2000	9	0.88 (0.80‒0.95)	0.50	0.89 (0.82‒0.97)	0.68
Before 2000	7	0.79 (0.67‒0.90)	.	0.84 (0.71‒0.97)	.

AUC, Area Under the ROC Curve; ADC, Apparent Diffusion Coefficient; BOLD, Blood Oxygen Level Dependent; CEUS, Contrast-Enhanced Ultrasound; DWI, Diffusion Weighted Imaging; DDS, Duplex Doppler Sonography; GTA, Grayscale Ultrasound Texture Analysis; NR, Not Reported PI, Pulsatility Index; PDI, Power Doppler Imaging; ROC, Receiver Operating Characteristic; RI, Resistive Index; SWE, Shear Wave Elastography; SWS, Shear Wave Speed.

Substantial between-study heterogeneity was observed, particularly in the US-based analyses (Fig. 8). Although pooled AUC values were statistically significant, the wide confidence regions and variability across studies indicate that these summary measures should be interpreted as reflecting a range of plausible diagnostic performance rather than a single precise estimate applicable to all clinical settings. In contrast, MRI-based parameters demonstrated more consistent effect directions across studies, with comparatively lower heterogeneity (Fig. 8), although the limited number of available studies precluded definitive conclusions. Overall, these findings highlight marked variability in diagnostic performance estimates, especially for US-based imaging, underscoring the influence of differences in study design, imaging protocols, and clinical context.

In subgroup analyses by imaging modality, pooled sensitivity, specificity, and AUC for MRI were 0.89 (95% CI 0.79‒0.95), 0.91 (95% CI 0.83‒0.95), and 0.96 (95% CI 0.94‒0.97), respectively (Fig. 8B). For US, pooled sensitivity, specificity, and AUC were 0.79 (95% CI 0.68‒0.88), 0.89 (95% CI 0.77‒0.95), and 0.90 (95% CI 0.88‒0.93), respectively (Fig. 8C).

Further meta-regression and subgroup analyses demonstrated that the reference standard (p = 0.02) and imaging examination interval (p = 0.02) were significant sources of heterogeneity for sensitivity, whereas no significant heterogeneity was observed for specificity. In the subgroup using biopsy alone as the reference standard, the pooled sensitivity was 0.78 (95% CI 0.63–0.92). In contrast, in studies using biopsy combined with treatment response as the reference standard, the pooled sensitivity was higher at 0.86 (95% CI 0.79–0.94). Regarding the imaging examination interval, studies with an interval ≤ 1-day showed a pooled sensitivity of 0.87 (95% CI 0.78–0.95), while those with an interval > 1-day demonstrated a comparable pooled sensitivity of 0.81 (95% CI 0.71–0.92) (Table 2).

### Heterogeneity analysis

**Fig. 5:**
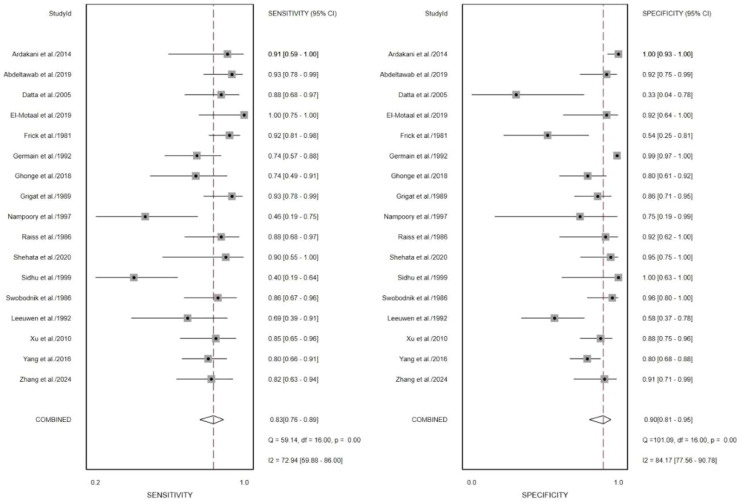
Forest plot of sensitivities and specificities of the included studies expect the study of Barquin^23^ et al.

**Fig. 6:**
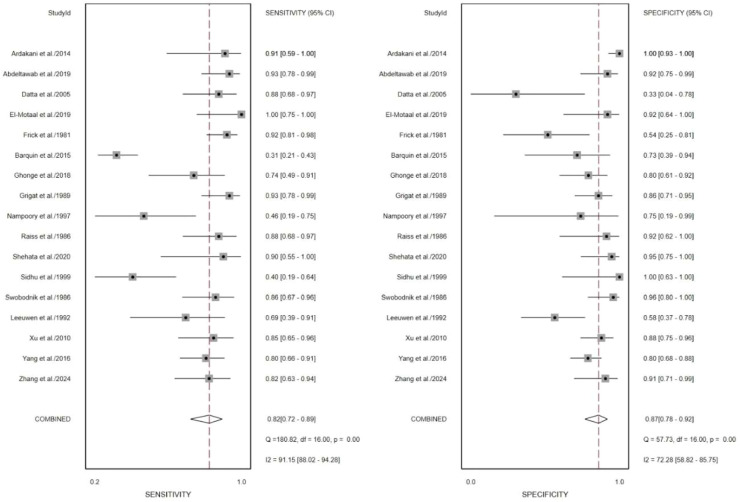
Forest plot sensitivities and specificities of the included studies expect the study of Germain^15^ et al.

**Fig. 7:**
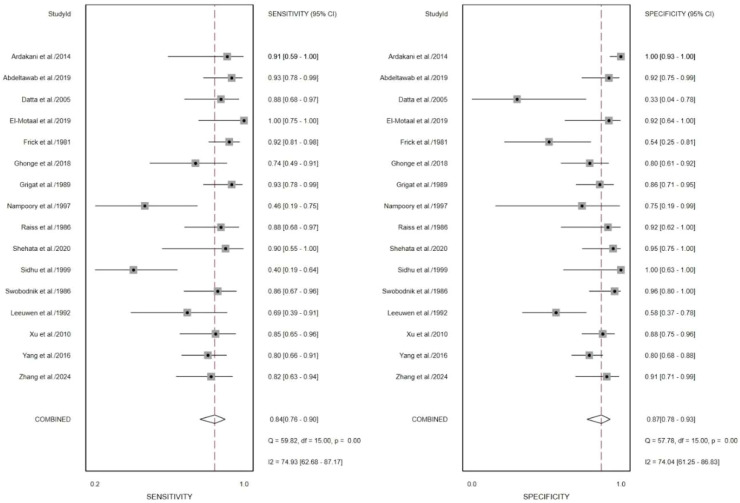
Forest plot sensitivities and specificities of the included studies expect the study of Barquin et al.^23^ and Germain^15^ et al.

The pooled I^2^ values for sensitivity and specificity were 90.31% and 83.15%, respectively (Fig. 4), indicating substantial heterogeneity among the included studies. Therefore, further analyses were conducted to explore potential sources of heterogeneity. Sensitivity analysis using a leave-one-out approach revealed that exclusion of study of Barquin et al.^23^ resulted in a notable reduction in heterogeneity for sensitivity, with *I*^2^ decreasing from 90.31% to 72.94% (Fig. 5). Similarly, exclusion of study of Germain et al.^15^ led to a reduction in heterogeneity for specificity, with *I*^2^ decreasing from 83.15% to 72.28% (Fig. 6). When both studies Barquin et al.^23^ and Germain et al.^15^ were excluded simultaneously, the pooled *I*^2^ values for sensitivity and specificity were 74.93% and 74.04%, respectively (Fig. 7).

### Publication bias

The Deeks funnel plot (Fig. 10) exhibited an approximately symmetrical distribution, indicating no significant evidence of publication bias among the included studies (p = 0.15).

## Discussion

In recent years, US and MRI have emerged as promising noninvasive modalities for evaluating renal function and structure, offering considerable clinical potential. In this meta-analysis, the authors included 18 studies comprising 1140 kidney transplant recipients and found that imaging-based approaches demonstrated overall good diagnostic performance for detecting AIR, with pooled sensitivity and specificity of 0.82 and 0.89, respectively (Fig. 4). These findings support the potential role of noninvasive imaging in the assessment of graft status after transplantation.

**Fig. 8:**
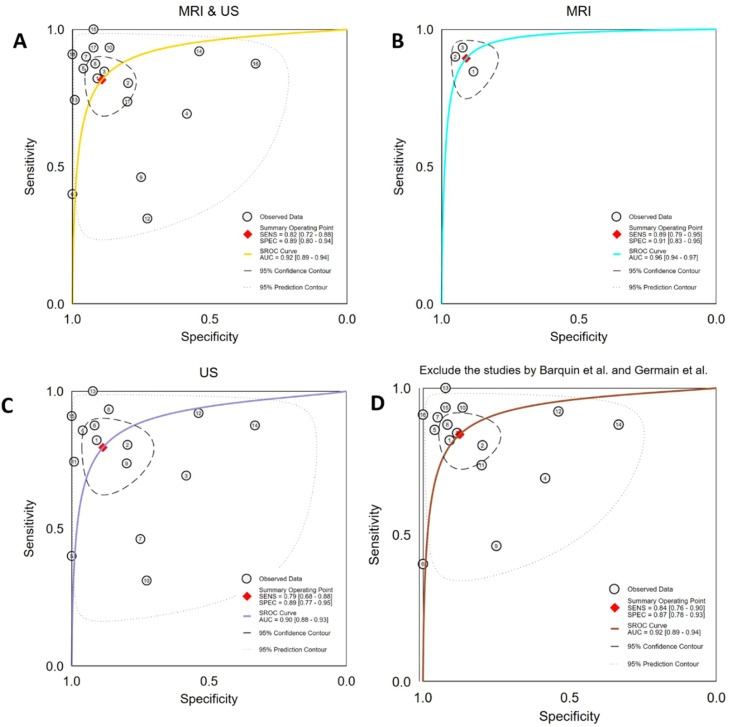
SROC curves for the included studies. (A) SROC curve for the included studies. (B) SROC curve for the MRI studies included. (C) SROC curve for the US studies included. (D) SROC curve for the included studies expect the study of Barquin et al.^23^ and Germain^15^ et al.

Given the fundamental differences in physiological mechanisms captured by MRI and US, the authors analyzed these modalities separately. In the MRI group (3-studies), the pooled AUC, sensitivity, and specificity were 0.96 (95% CI 0.94–0.97), 0.89, and 0.91, respectively (Fig. 8B). In the US group (15-studies), the pooled AUC, sensitivity, and specificity were 0.90 (95% CI 0.88–0.93), 0.79, and 0.89 (Fig. 8C), respectively.

Sensitivity analyses excluding two studies^15^^,^^23^ showed a substantial reduction in heterogeneity. sensitivity *I*^2^ changes form 90.31% to 74.93%, specificity I²changes form 83.15% to 74.04% (Fig. 7), suggesting that these studies were contributors to the observed variability. Several factors may explain this finding. First, both studies were based on the US, which is known to be highly operator-dependent and subject to variability in acquisition and interpretation. Differences in equipment, operator experience, and diagnostic criteria may have introduced additional heterogeneity.^26^, ^27^, ^28^, ^29^ Second, the study by Germain et al.^15^ was conducted in an earlier era of ultrasound technology, when imaging resolution and standardization were considerably limited compared to modern systems. This temporal gap may have led to systematic differences in diagnostic performance relative to more recent studies. Third, both studies employed retrospective designs, which are more prone to selection bias and inconsistencies in reference standards, further contributing to variability in reported diagnostic accuracy. Taken together, these factors suggest that differences in imaging modality characteristics, technological evolution, and study design may underlie the observed heterogeneity.

**Fig. 9:**
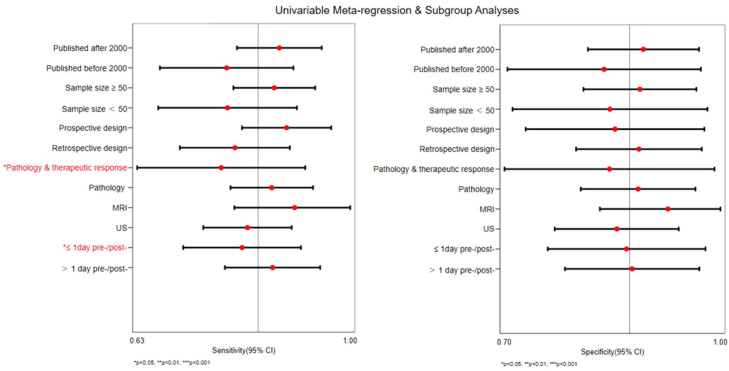
Univariable meta-regression and subgroup analyses.

Subsequently, Subgroup analyses indicated that both the reference standard and imaging examination interval were significant sources of heterogeneity (Fig. 9), suggesting that these factors may influence the estimated diagnostic performance.^32^ Specifically, among studies using a combined reference standard ‘pathology + therapeutic’ demonstrated higher diagnostic performance than those relying on histopathology alone, indicating that integrated clinical criteria may better reflect the underlying disease status. In addition, the interval between imaging examination and biopsy emerged as an important contributor to variability. Studies with an interval ≥1-day showed slightly lower sensitivity compared with those with an interval <1-day, suggesting that prolonged time gaps may reduce the concordance between imaging findings and histopathological results. This discrepancy is likely attributable to the dynamic and rapidly evolving nature of AIR, where pathological alterations may progress or partially regress within a short time frame, leading to temporal mismatches between imaging and biopsy-based assessments. In the early stages of AIR, the renal microenvironment undergoes a series of changes, including inflammatory cell infiltration, edema, microvascular perfusion abnormalities, and expansion of the extracellular space, all of which can be indirectly captured by different imaging parameters.^33^ For example, ADC typically decreases due to restricted water molecule diffusion caused by inflammatory infiltration and edema:^34^ the R2* value in BOLD often increases under ischemic and hypoxic conditions, indicating reduced tissue oxygenation.^35^ Doppler ultrasound indices such as Pulsatility Index (PI) and Resistive Index (RI) generally increase in response to elevated microvascular resistance, reflecting impaired perfusion.^32^ These findings highlight that the observed heterogeneity likely reflects a combination of methodological and technical variability rather than a single dominant source.

**Fig. 10:**
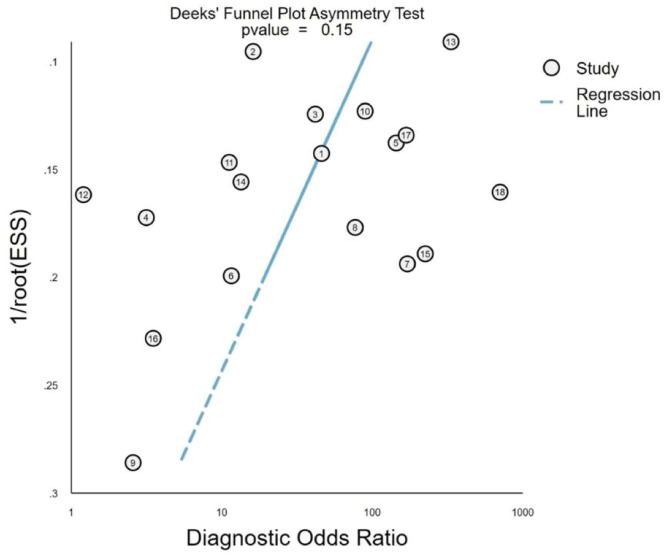
Funnel plot of the included studies. ESS, Effective Sample Size.

Several limitations should be acknowledged. First, the majority of included studies were retrospective, and many lacked prespecified thresholds or blinded assessment, introducing potential bias. Second, for studies reporting multiple imaging parameters, the authors selected the best-performing parameter, which may overestimate diagnostic performance. Third, multivariable meta-regression was not possible due to the limited number of studies, and the identified sources of heterogeneity should be interpreted with caution as they may be confounded. particularly for MRI, only three studies focusing on DWI and combined DWI & BOLD sequences were eligible; while their diagnostic accuracy could be estimated using a random-effects model, further validation will be required once more data become available. Parameters such as Fractional Anisotropy (FA) from DTI and the pseudo-diffusion coefficient (D*) and perfusion fraction (f) from IVIM also show potential for application,^9^^,^^36^, ^37^, ^38^ but current evidence is limited, and further validation is warranted.^39^ Fourth, the timing of imaging assessments varied substantially across studies, potentially undermining the stability of diagnostic parameters. Therefore, the present results should be interpreted as reflecting the upper-bound performance of imaging-based approaches rather than their expected real-world accuracy.^40^, ^41^, ^42^ Fifth, heterogeneity in imaging protocols, timing, and parameter definitions may affect the generalizability of the results. Finally, some studies did not distinguish between different subtypes of rejection, which may have different imaging characteristics.^43^

Clinically, these findings suggest that MRI and US may serve as useful adjunctive tools for noninvasive assessment of AIR, with potential applications in early screening, risk stratification, and longitudinal monitoring. However, given the observed heterogeneity and the lack of standardized imaging protocols and diagnostic thresholds, current evidence does not support replacing histopathological biopsy. Imaging should therefore be considered complementary to biopsy rather than a standalone diagnostic approach.

Future studies should focus on large-scale, prospective, multicenter designs with standardized imaging protocols and predefined diagnostic criteria.^44^ In addition, the development of multiparametric and model-based approaches,^20^^,^^45^ including radiomics and machine learning,^1^ may further improve diagnostic performance and support precision medicine in kidney transplantation.

## Conclusion

In conclusion, this meta-analysis demonstrates that both MRI and US show good diagnostic performance for detecting AIR after kidney transplantation. However, the currently available evidence remains limited in sample size and number of studies, and substantial heterogeneity exists across studies. Therefore, these imaging modalities should be considered adjunctive tools to histopathological biopsy rather than replacements for definitive diagnosis. Future large-scale, prospective, and multicenter studies with standardized imaging protocols are needed to further validate their clinical utility and to better define their role in routine post-transplant surveillance.

## Ethics approval and consent to participate

Not applicable.

## Consent for publication

Not applicable.

## Data availability

No datasets were generated or analyzed during the current study. Therefore, data sharing is not applicable to this article.

## Authors' contributions

Zhi-qiang Ouyang and Rong Qian conceived the study idea and designed the experiments. Xinyan Zhou and Yaifei Zhang performed the statistical analysis. Chende Liao, Shasha Bao, Na Tan, Wan Shen, Yihua Bai, Tao Wu, Guodong Zhang, Dong Chen, and Yi Lu prepared all figures and tables. Rong Qian wrote the first draft of the manuscript. All authors contributed to manuscript revision, read, and approved the submitted version.

## Funding

This work was supported by the Youth Project of Yunnan Provincial Science and Technology Department applied basic research (n° 202501AU070169), the Kunming Health Technology Talents Training Project (n° 2024-SW-51), the 10.13039/501100001809National Natural Science Foundation of China (n° 82160340), the Key Project of Yunnan Provincial Science and Technology Department applied basic research (n° 202501AY070001-039), the Yunnan Provincial Science and Technology Department & Kunming Medical University applied basic research (n° 202401AY070001-268) and the Yunnan Provincial Science and Technology Department & Kunming Medical University applied basic research (n° 202201AY070001-069).

## Conflicts of interest

The authors declare no conflicts of interest.

## Appendix Supplementary materials

Image, application 1
