# Supplementary material for: Diagnostic accuracy of MRI and US for identifying acute rejection after allogeneic kidney transplantation: a systematic review and meta-analysis
Source: Clinics (Sao Paulo). 2026 May 18;81:100999. doi: 10.1016/j.clinsp.2026.100999 (PMC13213319; doi:10.1016/j.clinsp.2026.100999)

**CLINICS-D-25-01620_Supplementary Material**

**Table S1** (continued Table 1) Basic characteristics of the included studies.

| **Nº** | **Image sequence** | **Quantitative parameter** | **Included case number (M/F)** | **AIR/non-AIR** | **TP** | **FP** | **TN** | **FN** | **Sensitivity** | **Specificity** | **Accuracy** | **Cut off** | **AUC** |
| --- | --- | --- | --- | --- | --- | --- | --- | --- | --- | --- | --- | --- | --- |
| 1 | Conventional US & CEUS | PI | 50/40/10 | 28/22 | 23 | 2 | 20 | 5 | 0.82 | 0.91 | 0.86 | 4132.76 | 0.86 |
| 2 | SWE & Doppler US | SWS&RI | 115/82/33 | 46/69 | 37 | 14 | 55 | 9 | 0.80 | 0.80 | 0.80 | SWS>2.90 | 0.80 |
| 3 | DWI | ADC | 69/NR/NR | 26/43 | 22 | 5 | 38 | 4 | 0.85 | 0.88 | 0.87 | ADC = 2.155 × 10^-3^ | 0.87 |
| 4 | DDS | PI | 37/22/15 | 13/24 | 9 | 10 | 14 | 4 | 0.69 | 0.58 | 0.62 | PI>1.4 + ΔPI>0.2 | 0.62 |
| 5 | Real-time B-mode ultrasound | NR | 50/34/16 | 28/25 | 24 | 1 | 24 | 4 | 0.86 | 0.96 | 0.91 | NR | 0.91 |
| 6 | PDI | NR | 28/17/11 | 20/8 | 8 | 0 | 8 | 12 | 0.40 | 1.00 | 0.57 | NR | 0.57 |
| 7 | DWI & BOLD | ADC & R2* | 30/NR/NR | 10/20 | 9 | 1 | 19 | 1 | 0.90 | 0.95 | 0.93 | NR | 0.93 |
| 8 | Real-time B-mode ultrasound | NR | 36/NR/NR | 24/12 | 21 | 1 | 11 | 3 | 0.88 | 0.92 | 0.89 | NR | 0.89 |
| 9 | Real-time B-mode ultrasound | NR | 17/NR/NR | 13/4 | 6 | 1 | 3 | 7 | 0.46 | 0.75 | 0.53 | NR | 0.53 |
| 10 | DDS | PI | 60/NR/NR | 30/37 | 28 | 5 | 32 | 2 | 0.93 | 0.86 | 0.90 | PI=1.70 | 0.90 |
| 11 | SWE & Doppler | SWS &RI | 49/NR/NR | 19/30 | 14 | 6 | 24 | 5 | 0.74 | 0.80 | 0.78 | NR | 0.78 |
| 12 | Doppler | RI | 85/64/21 | 74/11 | 23 | 3 | 8 | 51 | 0.31 | 0.73 | 0.36 | RI<0.55 or >0.75 | 0.36 |
| 13 | CDUS | RI | 268/NR/NR | 35/233 | 26 | 2 | 231 | 9 | 0.74 | 0.99 | 0.96 | RI>0.80 | 0.96 |
| 14 | Real-time B-mode ultrasound | NR | 63/NR/NR | 50/13 | 46 | 6 | 7 | 4 | 0.92 | 0.54 | 0.84 | NR | 0.84 |
| 15 | Real-time B-mode ultrasound & CDUS & SE | SWS & RI | 40/26/14 | 13/13 | 13 | 1 | 12 | 0 | 1.00 | 0.92 | 0.96 | ≥14.96 | 0.96 |
| 16 | Real-time B-mode ultrasound & DDS & PDS | RI& PI | 30/25/5 | 24/6 | 21 | 4 | 2 | 3 | 0.88 | 0.33 | 0.77 | NR | 0.77 |
| 17 | DWI | ADC | 56/38/18 | 30/26 | 28 | 2 | 24 | 2 | 0.93 | 0.92 | 0.93 | NR | 0.93 |
| 18 | GTA | NR | 61/40/21 | 11/50 | 10 | 0 | 50 | 1 | 0.91 | 1.00 | 0.98 | NR | 0.98 |

AUC, Area Under the ROC Curve; ADC, Apparent Diffusion Coefficient; BOLD, Blood Oxygen Level Dependent; CEUS, Contrast-Enhanced Ultrasound; DWI, Diffusion Weighted Imaging; DDS, Duplex Doppler Sonography; GTA, Grayscale ultrasound Texture Analysis; NR, Not Reported PI, Pulsatility Index; PDI, Power Doppler Imaging; ROC, Receiver Operating Characteristic; RI, Resistive Index; SWE, Shear Wave Elastography; SWS, Shear Wave Speed.

**Figure S1** Forest plot positive likelihood ratios and negative likelihood ratios of the included studies. DLR, diagnostic likelihood ratio.


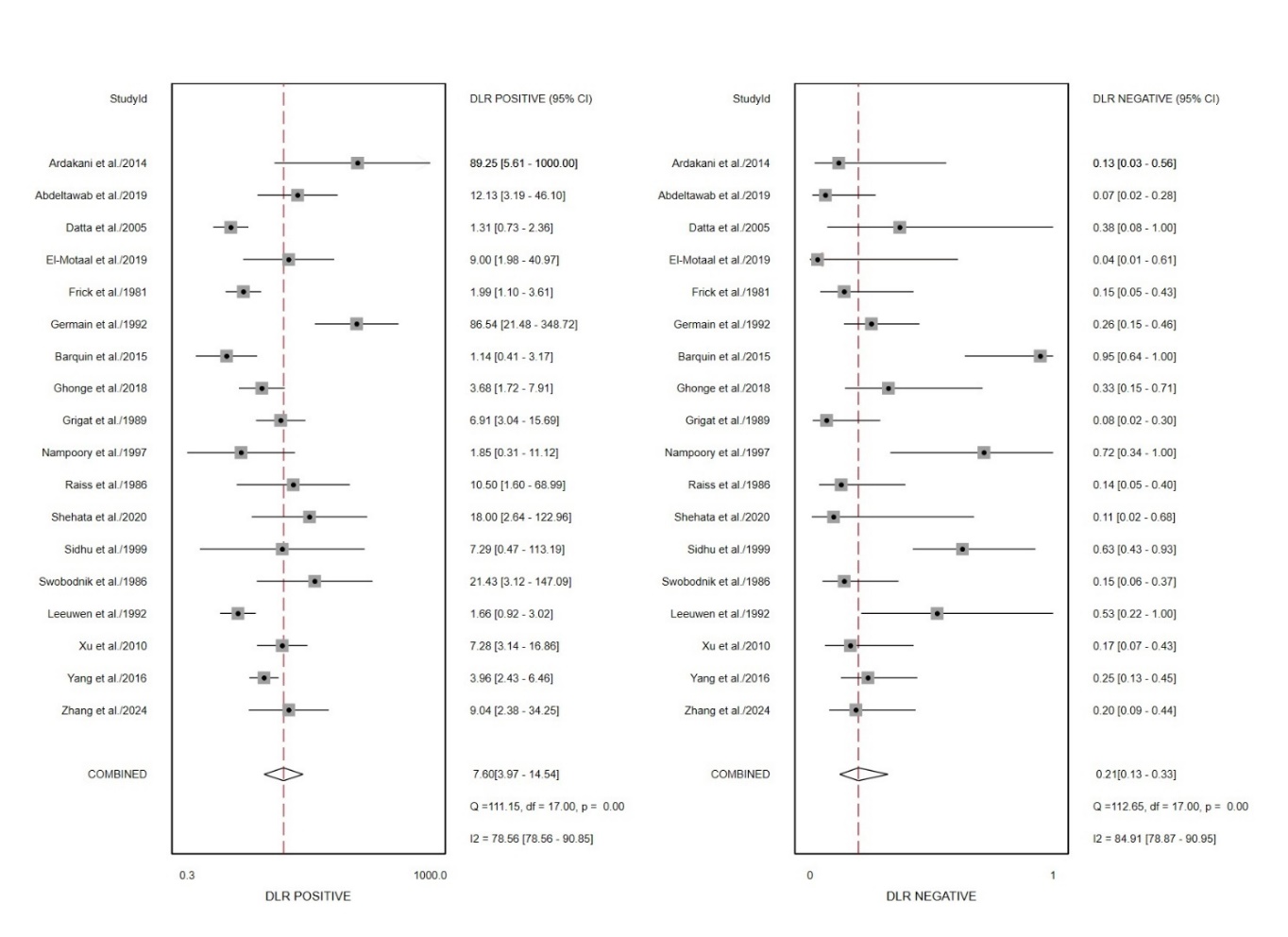


**Figure S2** Forest plot diagnostic score and odds ratio of the included studies.


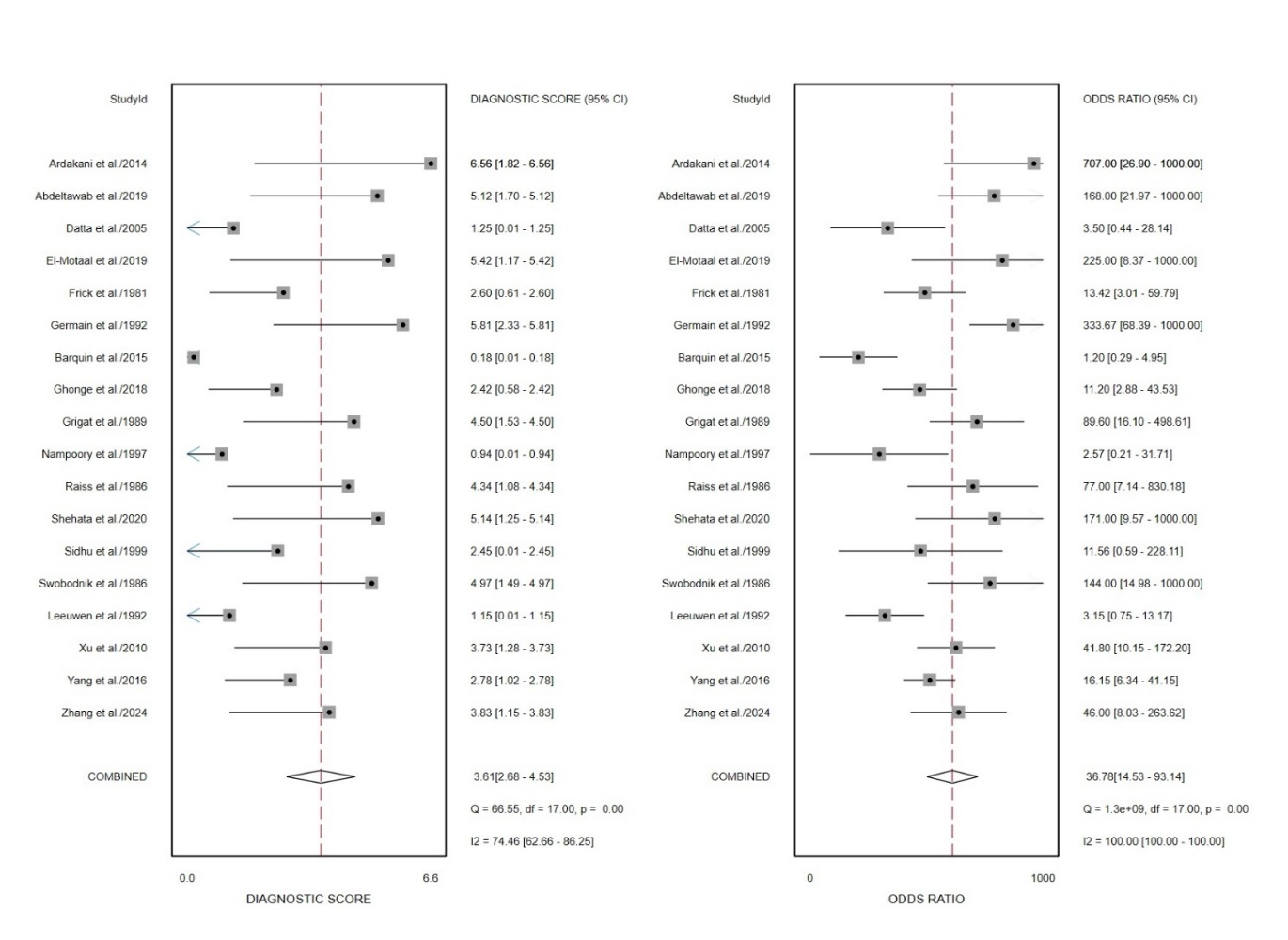


**Figure S3** Forest plot positive likelihood ratios and negative likelihood ratios of the included studies expect the study of Barquin et al. (2015)[23] and Germain et al. (1992).[15]


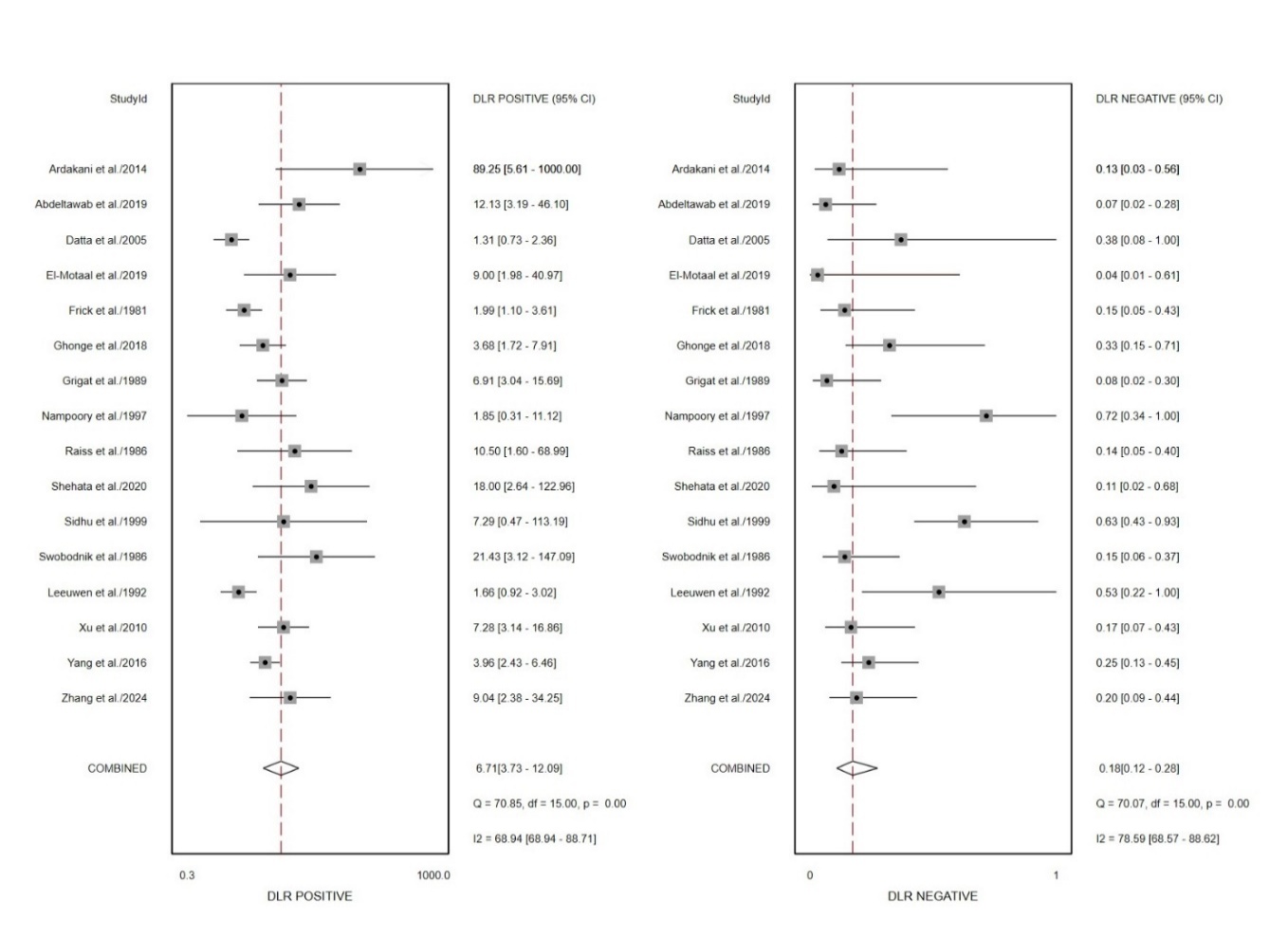


**Figure S4** Forest plot diagnostic score and odds ratio of the included studies expect the study of Barquin et al. (2015)[23] and Germain et al. (1992).[15]


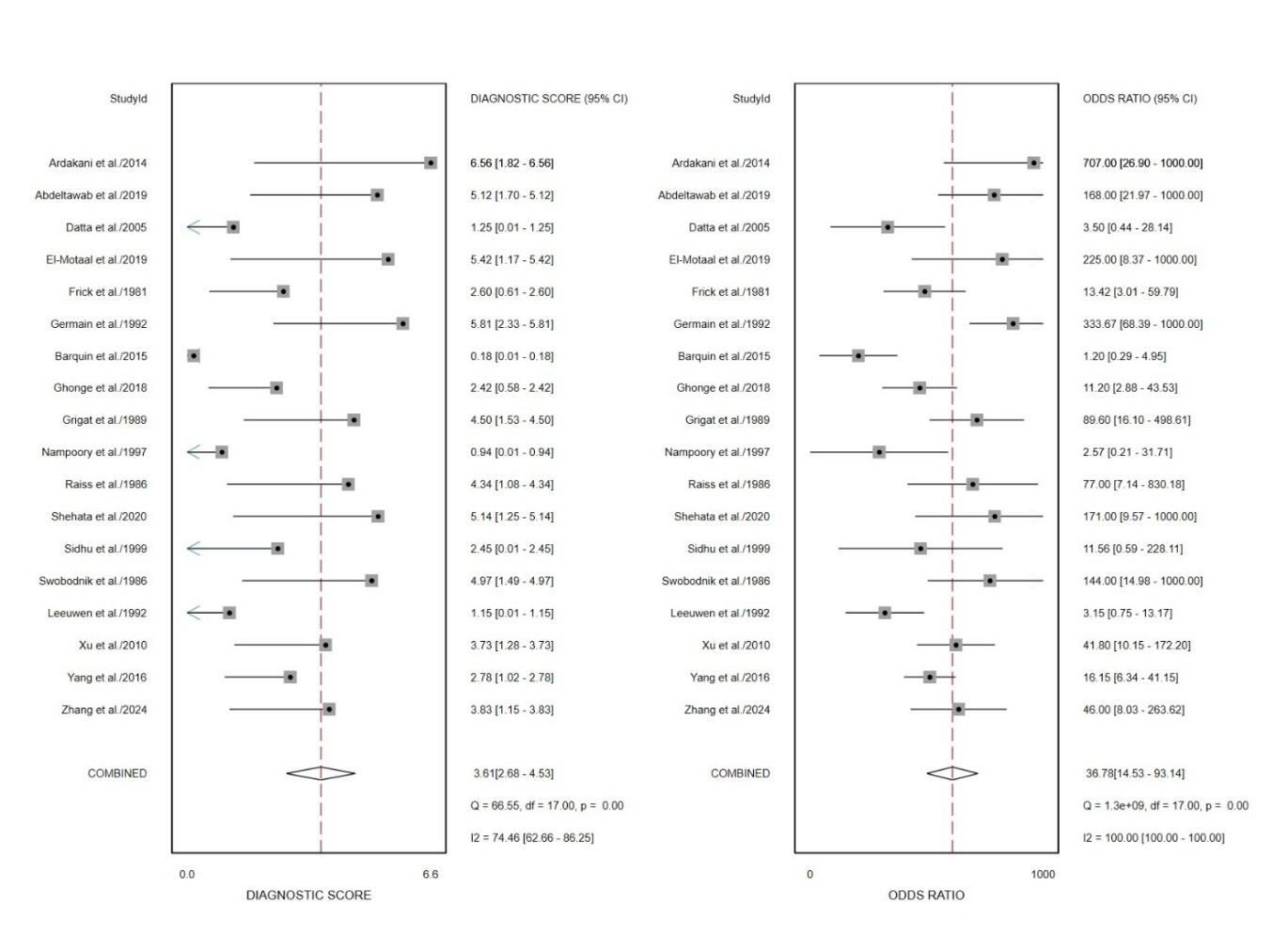

Supplement: Supplementary file 1 [file mmc1.docx]
